# Brain senescence drives sarcopenia-like transcriptomic remodeling in skeletal muscle

**DOI:** 10.1007/s11357-026-02205-y

**Published:** 2026-05-14

**Authors:** Shoba Ekambaram, Roland Patai, Rafal Gulej, Tamas Kiss, Siva Sai Chandragiri, Dorina Nagy, Kiana Vali Kordestan, Tamas Lakat, Stefano Tarantini, Peter Mukli, Andriy Yabluchanskiy, Anna Ungvari, Zoltán Benyó, Anna Csiszar, Zoltan Ungvari

**Affiliations:** 1 Vascular Cognitive Impairment and Neurodegeneration Program, Oklahoma Center for Geroscience and Healthy Brain Aging, Department of Neurosurgery, University of Oklahoma Health Sciences Center, Oklahoma City, OK USA; 2 Oklahoma Center for Geroscience and Healthy Brain Aging, University of Oklahoma Health Sciences Center, Oklahoma City, OK USA; 3 International Training Program in Geroscience, Doctoral College, Health Sciences Program/Institute of Preventive Medicine and Public Health, Semmelweis University, Budapest, Hungary; 4 Pediatric Center, MTA Center of Excellence, Semmelweis University, Budapest, Hungary; 5 Neurocognitive Diseases Research Group, HUN-REN-SU Cerebrovascular, 1094 Budapest, Hungary; 6 Healthy Aging Program, Institute for Translational Research, Budapest, Hungary; 7 Diabetes Research Group, MTA-SE Lendület Momentum, Budapest, Hungary; 8 Fodor Center for Prevention and Healthy Aging, Semmelweis University, Budapest, Hungary; 9 The Peggy and Charles Stephenson Cancer Center, University of Oklahoma Health Sciences Center, Oklahoma City, OK USA; 10 Institute of Preventive Medicine and Public Health, Semmelweis University, Budapest, Hungary; 11 Institute of Clinical Pathophysiology, Semmelweis University, Budapest, Hungary

**Keywords:** Senescence, Systemic aging, Cell non-autonomous aging, Skeletal muscle aging, Sarcopenia, Mitochondrial dysfunction, Accelerated aging, Senescence-associated secretory phenotype (SASP), Irradiation, Transcriptomics, Frailty, FOXO signaling

## Abstract

Aging is accompanied by a progressive decline in skeletal muscle mass and function, culminating in sarcopenia, a major contributor to frailty, disability, and mortality in older adults. While skeletal muscle aging has traditionally been attributed to cell-autonomous and local tissue mechanisms, increasing evidence suggests that systemic, cell non-autonomous processes play a central role in coordinating aging across organs. The brain, particularly the hypothalamus, has emerged as a key regulator of organismal aging, yet its contribution to skeletal muscle aging remains poorly defined. Here, we tested the hypothesis that senescence confined to the brain is sufficient to induce aging-like molecular remodeling in skeletal muscle via systemic mechanisms. To model brain senescence, young mice were subjected to fractionated whole-brain irradiation (WBI), a well-established approach that induces widespread cellular senescence and neuroinflammation in the brain while sparing peripheral tissues. Two months after WBI, transcriptomic profiling of quadriceps muscle was performed and compared with that of naturally aged mice. WBI-induced robust gene expression changes in skeletal muscle that closely mirrored those observed during chronological aging. Pathway-level analyses revealed marked downregulation of mitochondrial organization, respiratory chain assembly, and metabolic processes, alongside enrichment of remodeling- and stress-associated pathways. Upstream regulator analysis identified FOXO1, FOXO3, KLF15, and STAT3, which are key drivers of muscle catabolism and atrophy, as central mediators of the observed transcriptional program. Semantic similarity analysis further demonstrated a high concordance between WBI-induced and aging-associated biological processes. Collectively, these findings demonstrate that brain senescence is sufficient to drive sarcopenia-like transcriptomic remodeling in skeletal muscle, implicating central nervous system aging as an upstream regulator of peripheral muscle decline. This brain–muscle aging axis may contribute to frailty in individuals with accelerated brain aging and in cancer survivors exposed to cranial irradiation, highlighting brain senescence as a potential therapeutic target to mitigate systemic aging and skeletal muscle dysfunction.

## Introduction

Aging is accompanied by a progressive decline in skeletal muscle mass, strength, and metabolic capacity, a condition clinically recognized as sarcopenia [1, 2]. Sarcopenia represents a major driver of frailty, disability, loss of independence, and mortality in older adults and contributes substantially to the societal and healthcare burden of aging populations. Although skeletal muscle aging has traditionally been attributed to local, cell-autonomous mechanisms (e.g. mitochondrial dysfunction [3, 4], impaired proteostasis, altered satellite cell function, and chronic low-grade inflammation [5, 6]), it has become increasingly clear that muscle aging unfolds in concert with aging of other organs, suggesting the presence of higher-order systemic regulators [7–11].

A growing body of evidence supports the concept that aging is, at least in part, coordinated by cell non-autonomous mechanisms [7]. Inter-organ communication via circulating factors, neuroendocrine signaling, and immune mediators plays a critical role in synchronizing aging trajectories across tissues [7]. Landmark studies using heterochronic parabiosis and plasma transfer models have demonstrated that exposure of young animals to an aged systemic milieu is sufficient to induce aging-like phenotypes in skeletal muscle, including impaired regeneration, mitochondrial dysfunction, and increased expression of atrophy-related genes [8–12]. These findings implicate blood-borne pro-geronic signals as powerful modulators of muscle aging. However, the tissue sources and upstream regulators of these systemic aging cues remain incompletely understood.

The brain, and particularly the hypothalamus, has emerged as a central regulator of organismal aging [13–18]. As a neuroendocrine and homeostatic hub, the hypothalamus integrates metabolic, hormonal, inflammatory, and environmental signals to maintain systemic equilibrium throughout life [18–24]. Age-related hypothalamic dysfunction has been linked to diverse aging phenotypes, including metabolic dysregulation, impaired stress resilience, and physiological decline in multiple organ systems [13, 15, 16, 25]. Mechanistically, brain aging is characterized by chronic sterile inflammation [7, 26, 27], disruption of neuroendocrine signaling [15, 16, 19–21, 23, 28, 29], and accumulation of senescent cells, particularly among microglia and brain microvascular endothelial cells, which adopt a senescence-associated secretory phenotype (SASP) [30–34]. These senescent cells release pro-inflammatory cytokines and extracellular vesicles that may enter the circulation and influence peripheral tissues.

Recent studies have established that brain senescence contributes to neuroinflammation, blood–brain barrier dysfunction, and cognitive decline [30, 35]. However, whether increased presence of senescence cells in the brain can actively drive aging-like remodeling in peripheral tissues such as skeletal muscle remains largely unexplored. This represents a critical gap in our understanding of systemic aging, particularly given the high sensitivity of skeletal muscle to endocrine, inflammatory, and metabolic cues. Skeletal muscle mass and function are tightly regulated by circulating anabolic factors, including insulin-like growth factor-1 (IGF-1) [36–40], as well as by inflammatory mediators [41, 42] and stress-responsive signaling pathways. Age-associated disruption of these systemic inputs contributes to anabolic resistance, mitochondrial dysfunction, and activation of catabolic transcriptional programs that promote muscle atrophy. Experimental models in which the systemic environment of young animals is chronically altered by exposure to aged circulation have shown that age-associated circulating signals can impair muscle integrity and function [8, 10, 11, 43–45], while also affecting other peripheral tissues such as the heart and the vasculature [46, 47]. These observations underscore the ability of soluble, blood-borne factors to transmit aging phenotypes across organs and individuals.

Importantly, clinical observations support a link between central nervous system injury and peripheral muscle decline. Cancer survivors who undergo cranial irradiation frequently exhibit long-term endocrine dysfunction [48–54], reduced circulating IGF-1 levels, and accelerated onset of frailty-related phenotypes, including muscle weakness and reduced physical performance [55–62]. Cranial irradiation is known to induce long-lasting cellular senescence in multiple brain cell types, including astrocytes, microglia, and vascular endothelial cells, resulting in chronic neuroinflammation and sustained release of senescence-associated secretory factors [63–65]. These findings raise the possibility that brain-targeted insults that induce senescence and neuroinflammation may exert systemic pro-aging effects on skeletal muscle, independent of direct muscle injury.

In the present study, we tested the hypothesis that senescence induced selectively in the brain is sufficient to promote aging-like molecular remodeling in skeletal muscle via systemic, cell non-autonomous mechanisms. To this end, we employed a well-characterized model of fractionated whole-brain irradiation (WBI) in young mice, which induces widespread hypothalamic senescence [63] while sparing peripheral tissues from direct radiation exposure. Using transcriptomic profiling of quadriceps muscle, we compared WBI-induced molecular changes with those observed during natural aging. Our findings demonstrate that brain senescence drives a sarcopenia-like transcriptional program in skeletal muscle, characterized by mitochondrial dysfunction, metabolic suppression, and activation of catabolic regulatory networks. These findings demonstrate that brain senescence is sufficient to recapitulate core features of skeletal muscle aging and identify the brain as an upstream orchestrator of peripheral aging. While prior studies using heterochronic parabiosis and plasma transfer models demonstrated that systemic factors can transmit aging phenotypes [7, 12, 43, 66–68], the upstream tissue sources responsible for generating these signals have remained incompletely defined. Our results provide direct experimental evidence that senescence in the brain can initiate systemic aging-like remodeling in skeletal muscle, supporting the existence of a brain–muscle aging axis with potential relevance to frailty in aging and in individuals exposed to senescence-inducing brain insults.

## Methods

### Experimental animals

**Fig. 1:**
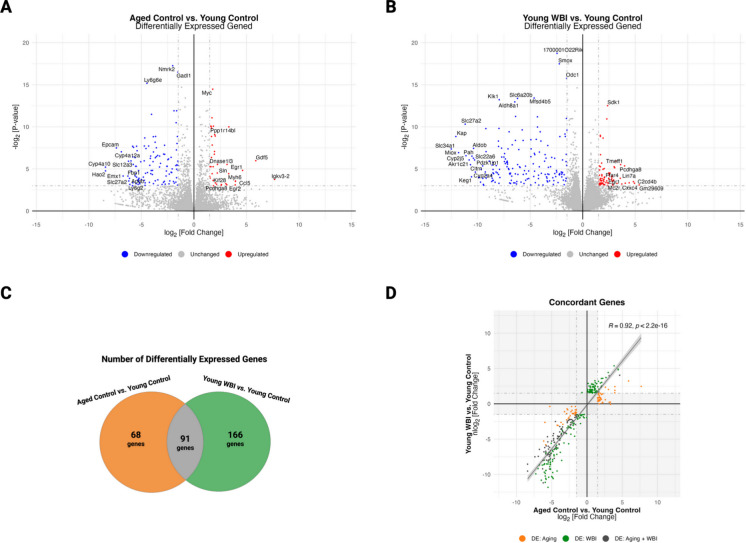
Whole-brain irradiation induces aging-like transcriptomic changes in skeletal muscle. **A** Volcano plot showing differentially expressed genes (DEGs) in the skeletal muscle of aged control mice compared with young control mice. **B** Volcano plot showing DEGs in the skeletal muscle of young mice 2 months after whole-brain irradiation (WBI), compared with age-matched young controls. **C** Venn diagram illustrating the number of DEGs identified in each comparison and their overlap. **D** Scatter plot demonstrating that, beyond sharing overlapping DEGs, gene expression changes are strongly positively correlated between aging and WBI-induced conditions. In addition to overlapping DEGs, many genes exhibited concordant directions of change despite being differentially expressed in only one comparison; these genes are highlighted in white in the scatter plot

Young (7 month old, *n* = 17) and aged (19 month old, *n* = 9) male C57BL/6 mice (*Mus musculus*) were obtained from the National Institute on Aging (NIA) colony and housed in a specific pathogen-free animal facility at the University of Oklahoma Health Sciences Center (OUHSC). Animals were maintained under a 12:12 h light–dark cycle with ad libitum access to standard rodent chow (AIN-93G) and water in accordance with standard husbandry practices. One week prior to the first brain irradiation session, all mice were transferred from the Rodent Barrier Facility to the Conventional Rodent Facility at OUHSC, where they were housed under similar conditions. The same cohort of animals was used for parallel analyses of multiple peripheral organs in related studies, enabling integrated assessment of systemic effects of brain senescence [63]. Nine young mice were randomly assigned to receive fractionated whole-brain irradiation (WBI; two sessions per week for 4 weeks, Fig. 1A). Following completion of the WBI protocol, irradiated mice were allowed to recover for 2 months. At the end of the recovery period, animals were humanely euthanized, and heart tissues were collected for transcriptomic analyses. Heart tissues from control mice were collected at 10 months (young) and 22 months (aged). All animal procedures were approved by the Institutional Animal Care and Use Committee (IACUC) at OUHSC and conducted in accordance with the National Institutes of Health Guide for the Care and Use of Laboratory Animals.

### Brain irradiation

Following 1 week of acclimation in the conventional facility, young mice were randomly assigned to either the WBI (*n* = 8) or control group (*n* = 9). During the irradiation procedures, mice were sedated using isoflurane anesthesia (Isoflurane USP, Cat# 029405, Covetrus, UK; 2.5% at a flow rate of 2.0 L/min). WBI was administered using an X-RAD320 X-ray irradiator (PRECISION, CT, USA) operating at 320 keV acceleration voltage and a dose rate of 0.8 Gy/min. The X-RAD 320 system is equipped with a collimator, which allows precise delivery of irradiation specifically to the brain, preventing off-target exposure to peripheral tissues. The WBI protocol consisted of eight irradiation sessions, delivered twice weekly over 4 weeks. Each session delivered a dose of 5 Gy, resulting in a cumulative total of 40 Gy. Following the final irradiation, mice were allowed to recover for 2 months.

### Tissue harvesting

At the experimental endpoint, animals were anesthetized with isoflurane and euthanized via intracardiac perfusion with ice-cold phosphate-buffered saline (PBS). Skeletal muscle tissue of quadriceps muscle from age-matched control mice was collected at 10 months of age (young controls) and 22 months of age (naturally aged controls). The dissected skeletal muscle tissue was then flash-frozen in liquid nitrogen and stored at –80 °C until further processing for transcriptomic analysis.

### RNA isolation, cDNA library preparation, and next-generation sequencing

Total RNA was isolated from frozen left ventricular heart tissue samples using the RNeasy Fibrous Tissue Mini Kit (cat#: 74,704, QiaGEN, Germany) according to the manufacturer’s instructions. Briefly, 30 mg of tissue was homogenized in RLT lysis buffer using mechanical disruption. The lysates were centrifuged at 1000 g for 10 min at 4 °C, and the resulting supernatants were transferred to clean microcentrifuge tubes. RNA was then isolated using the QIAcube Connect MDx system (QIAGEN, Germany), a fully automated platform utilizing spin column–based extraction technology. The quantity and integrity of the extracted RNA were assessed using both a NanoDrop Microvolume Spectrophotometer and an Agilent TapeStation system. The mean RNA Integrity Number (RIN) values were as follows: Young = 7.3 ± 1.3, Young + WBI = 8.9 ± 0.3, and Aged = 7.9 ± 1.4 (mean ± SD), indicating optimal RNA quality for downstream transcriptomic analysis.

Following quality assessment, cDNA library preparation was performed at the OUHSC Genomics Core Facility. Messenger RNA was enriched using the NEBNext Poly(A) mRNA Magnetic Isolation Module (Cat# E7490S, New England Biolabs, USA), and libraries were constructed using the xGen Broad Range RNA Library Prep Kit (Cat# 10,009,866, Integrated DNA Technologies, USA) in accordance with the manufacturer’s protocol. Library quality and concentration were verified using the Agilent TapeStation. Equimolar amounts of indexed libraries were pooled and sequenced on an Illumina NextSeq 2000 platform using sequencing-by-synthesis chemistry to generate 151 bp paired-end reads.

### RNA-Seq data processing and differential expression analysis

Raw sequencing reads were assessed for quality using FastQC (v0.11.9) and MultiQC (v1.12) to generate an aggregated quality report. Adapter sequences and low-quality reads (Phred score < 20) were removed using Trimmomatic (v0.39). Transcript abundance was quantified by pseudo-alignment to the mouse transcriptome (GRCm39) using Kallisto (v0.46.2).

Sample-level quality control and outlier detection were performed after quantification. Estimated counts and transcript lengths were imported and summarized to the gene level using the tximport R package (v1.38.2). Lowly expressed genes were filtered out, retaining only genes with at least 10 raw counts in at least 3 samples. Principal component analysis (PCA) was performed to assess sample clustering and identify potential technical outliers; however, all samples exhibited high biological consistency, and none was excluded from the final analysis.

All statistical analyses were performed using established RNA-seq analysis pipelines.

Differential expression analysis was performed using the DESeq2 R package (v1.50.2). DESeq2 utilizes a negative binomial generalized linear model (GLM) to model count data and Wald statistics for hypothesis testing. The raw counts, imported via tximport, were directly used as input. Multiple testing correction was performed using the Benjamini-Hochberg false discovery rate (FDR) method. Significantly differentially expressed (DE) genes were identified based on an FDR-adjusted *P* values < 0.05 and an absolute logarithmic fold change (|FC|) > 1.5. Gene annotations (Entrez IDs and gene symbols) were retrieved from Ensembl IDs using the org.Mm.eg.db R package (v3.22.0) for comprehensive annotation querying from the Ensembl database.

### Functional enrichment analysis

Gene Ontology (GO) terms (biological process, molecular function, cellular component), KEGG pathways, Reactome pathways, and Hallmark pathways associated with the differentially expressed gene sets were collected and analyzed. Gene set enrichment analysis (GSEA) was performed using the clusterProfiler R package (v4.18.4). For GSEA, genes were ranked by their weighted log2 Fold Change (FC * − log10(p)) values. Enrichment maps for visualizing the relationships between enriched pathways were created using the EnrichmentMap plugin (v3.5.0) within Cytoscape (v3.10.4). GO enrichment semantic similarity between aging and WBI effect was computed using the GOSemSim package (v2.36.0) with the clusterSim() function and default parameters [69].

### Upstream regulator analysis

Changes in transcription factor (TF) activity were predicted using the decoupleR R package (v2.8.0) by leveraging the DoRothEA mouse regulon [70]. Results of decoupleR were also validated by upstream regulator analysis available in the Ingenuity Pathway Analysis (IPA, QIAGEN) software. IPA is a widely used commercial bioinformatics platform that integrates differentially expressed genes with known biological functions, signaling pathways, and regulatory networks curated in the Ingenuity Knowledge Base. The URA algorithm predicts potential upstream transcriptional regulators based on the direction and magnitude of observed gene expression changes, providing activation *z*-scores and overlap *P* values to estimate the likelihood and directionality of regulator activity. Additional details on IPA methodology are available at: http://qiagen.force.com/KnowledgeBase/

## Results

### WBI accelerates aging-associated transcriptomic changes in skeletal muscle

To determine whether WBI induces aging-like molecular alterations in peripheral tissues, we analyzed gene expression profiles in skeletal muscle following WBI and compared them with those observed during chronological aging. WBI resulted in 257 differentially expressed genes (DEGs) in the skeletal muscle of young mice relative to age-matched controls (Fig. 1A). In parallel, chronological aging was associated with 159 DEGs when aged control mice were compared with young controls (Fig. 1B). Importantly, all 91 genes shared between the two DEG sets were regulated in the same direction in both aging and WBI conditions, indicating a strong overlap between WBI-induced and aging-associated transcriptional changes (Fig. 1C).

Beyond shared gene identity, the magnitude and direction of expression changes exhibited a strong positive correlation between WBI and aging (Pearson’s *R* = 0.92, *P* < 2.2 × 10⁻^1^⁶), demonstrating that WBI induces highly concordant transcriptomic responses rather than merely overlapping gene sets (Fig. 1D). Together, these findings indicate that whole-brain irradiation accelerates aging-like transcriptomic remodeling in skeletal muscle.

### WBI induces gene expression changes in skeletal muscle consistent with mitochondrial dysfunction and skeletal muscle atrophy

**Fig. 2:**
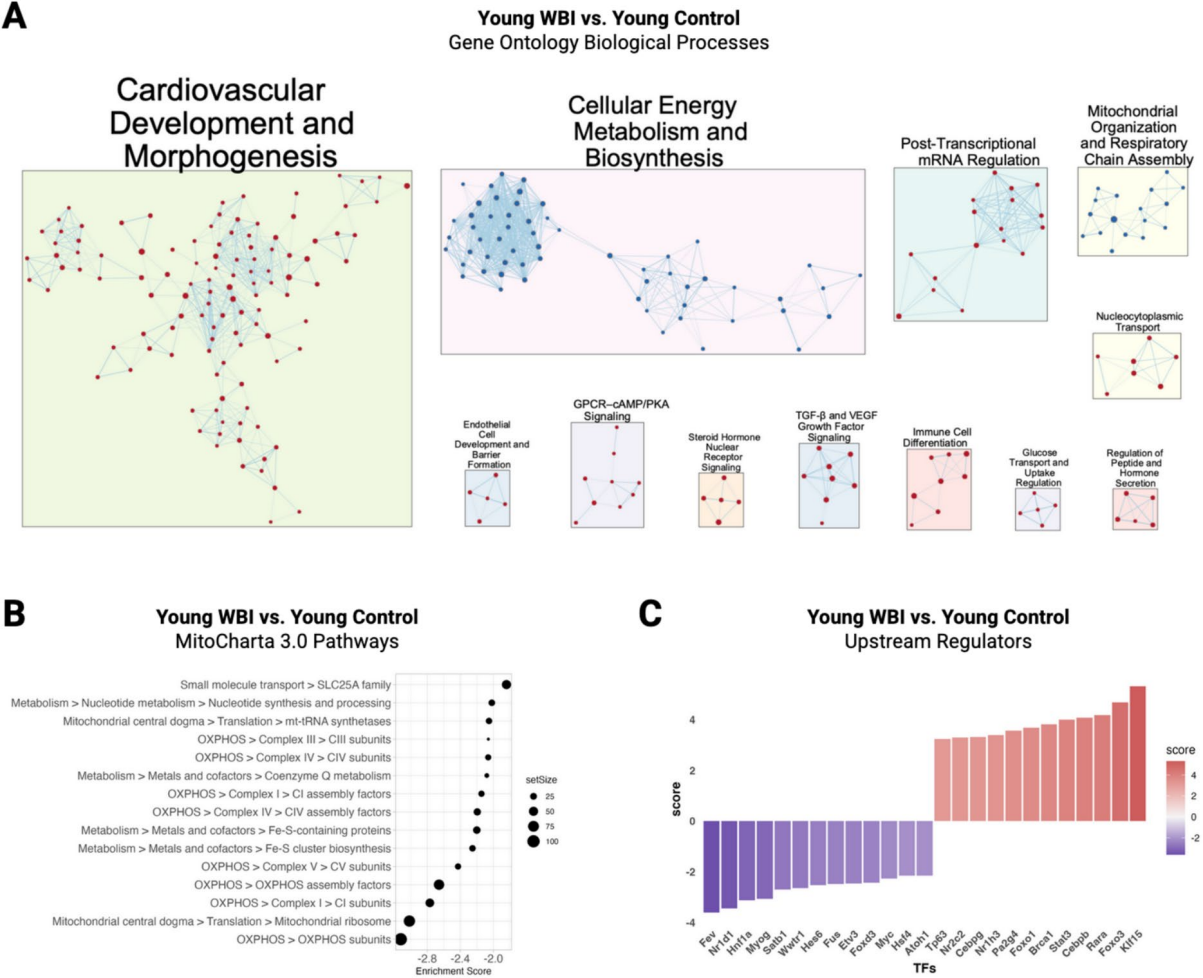
Whole-brain irradiation induces mitochondrial dysfunction. **A** Enrichment map visualization of Gene Ontology Biological Process gene set enrichment analysis (GSEA) in skeletal muscle following whole-brain irradiation. Red nodes indicate positively enriched (activated) pathways, whereas blue nodes indicate negatively enriched (downregulated) pathways. **B** MitoCarta 3.0–based gene set enrichment analysis highlighting alterations in mitochondrial pathways in skeletal muscle after whole-brain irradiation. Our plot shows the 15 downregulated pathways. **C** Upstream regulator analysis identifying predicted transcription factors associated with the observed gene expression changes. FOXO and KLF15 are central regulators of proteolysis, mitochondrial turnover, and muscle atrophy, supporting the interpretation that WBI induces a sarcopenia-like transcriptional state

To characterize transcriptomic alterations in skeletal muscle following WBI, gene set enrichment analysis (GSEA) was performed on ranked gene lists derived from WBI samples using Gene Ontology (GO) biological process annotations. Pathways related to cardiovascular development and morphogenesis were positively enriched, whereas gene sets associated with cellular metabolism, biosynthesis, mitochondrial organization, and respiratory chain assembly were negatively enriched (Fig. 2A). To further interrogate mitochondrial-specific effects, a targeted pathway analysis using the MitoCarta 3.0 database revealed coordinated downregulation of 15 mitochondrial pathways (Fig. 2B).

To gain insight into upstream regulatory mechanisms underlying these transcriptional changes, transcription factor activity was inferred from the observed gene expression patterns. Upstream regulator analysis identified KLF15, FOXO3, FOXO1, and STAT3 among the top predicted transcriptional regulators, all of which are well-established drivers of skeletal muscle catabolism and atrophy (Fig. 2C).

To determine whether WBI-induced transcriptional alterations reflected broader aging-related pathway-level changes rather than isolated gene effects, semantic similarity analysis of enriched GO terms was performed. This analysis yielded a similarity score of 0.871, indicating strong concordance between WBI-induced and aging-associated biological processes.

## Discussion

**Fig. 3:**
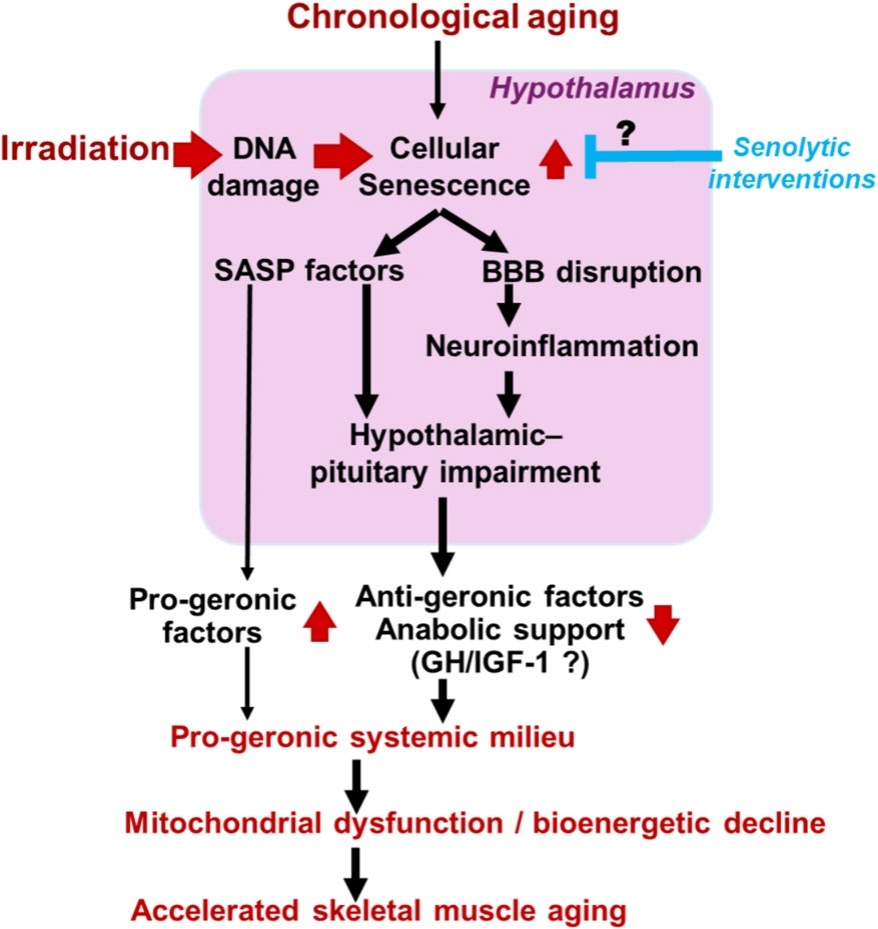
Proposed framework linking brain senescence to accelerated skeletal muscle aging. This schematic summarizes a working model in which aging-associated or irradiation-induced senescence within the brain, particularly affecting hypothalamic regulatory circuits, reshapes systemic signaling in a manner that promotes skeletal muscle aging. Chronological aging and whole-brain irradiation (WBI) increase DNA damage and senescent cell burden in the brain, leading to sustained inflammatory signaling and disruption of blood–brain barrier integrity. Senescent brain cells, including microglia, astrocytes, and cerebrovascular endothelial cells, generate a senescence-associated secretory phenotype (SASP) that enhances neuroinflammation and contributes to the release of pro-aging mediators into the circulation. At the same time, brain senescence interferes with hypothalamic–pituitary function, resulting in reduced neuroendocrine support and diminished anabolic signaling, potentially involving the growth hormone (GH)/insulin-like growth factor-1 (IGF-1) axis. The imbalance between elevated pro-geronic signals and reduced anti-geronic, anabolic factors establishes a systemic environment that favors mitochondrial dysfunction and bioenergetic decline in skeletal muscle. Through these converging cell non-autonomous pathways, brain senescence is proposed to accelerate sarcopenia-like molecular remodeling and functional deterioration of skeletal muscle. Potential therapeutic modulation of this axis, including brain-targeted senolytic strategies, is indicated as a hypothetical intervention point

The present study provides direct evidence that increased senescence burden in the brain is sufficient to induce aging-like molecular remodeling in skeletal muscle. Using a well-characterized model of fractionated WBI in young mice, we demonstrate that brain senescence drives a transcriptional program in skeletal muscle that closely mirrors that observed during chronological aging. The concordance between WBI-induced and age-associated gene expression changes supports the concept that skeletal muscle aging is strongly influenced by systemic, cell non-autonomous mechanisms originating in the central nervous system (Fig. 3).

A key finding of this study is the correlation between WBI-induced and aging-associated transcriptomic changes in skeletal muscle. Many overlapping differentially expressed genes were regulated in the same direction, and pathway-level analyses revealed highly concordant biological processes. Moreover, the magnitude and directionality of gene expression changes appeared to parallel those observed during chronological aging, indicating that WBI recapitulates physiological aging programs rather than inducing fundamentally distinct or exaggerated molecular responses. This level of alignment suggests that brain senescence does not merely perturb isolated molecular pathways in muscle but recapitulates the broader biological logic of muscle aging. These findings extend earlier observations from heterochronic parabiosis and plasma transfer studies, which demonstrated that exposure to an aged systemic milieu is sufficient to impair muscle regeneration and promote atrophy [8, 9, 43, 44, 71]. Our data identify brain senescence as a plausible upstream source of these systemic pro-geronic cues.

Functional enrichment analyses revealed that WBI-induced brain senescence results in suppressed mitochondrial organization, respiratory chain assembly, and metabolic pathways in skeletal muscle. Mitochondrial dysfunction is a hallmark of sarcopenia and contributes to reduced oxidative capacity, impaired ATP production, and increased susceptibility to fatigue [3, 4, 72–76]. The coordinated downregulation of mitochondrial gene sets observed here closely resembles transcriptional signatures reported in aged skeletal muscle and provides a mechanistic link between brain senescence and the energetic decline characteristic of muscle aging. These findings suggest that central nervous system aging can indirectly compromise muscle bioenergetics, potentially lowering the threshold for functional decline and frailty (Fig. 3).

In parallel with mitochondrial suppression, upstream regulator analysis identified transcription factors such as FOXO1 [77–80], FOXO3 [81–85], KLF15 [86–88], and STAT3 [89–91] as potential mediators of the observed transcriptional response. These factors are well-established regulators of muscle catabolism, proteolysis, and atrophy and are activated in diverse models of sarcopenia, cachexia, and disuse-induced muscle loss. FOXO signaling promotes expression of atrogenes and autophagy-related genes, while KLF15 regulates amino acid metabolism and muscle wasting under catabolic conditions. STAT3 activation has been linked to inflammatory muscle atrophy and impaired regeneration. The convergence of these regulators in WBI-exposed muscle strongly supports the interpretation that brain senescence induces a sarcopenia-like molecular program in skeletal muscle.

The mechanisms linking brain senescence to peripheral muscle aging are likely multifactorial (Fig. 3) with senescence-associated secretory signaling representing a central pathway. Multiple brain cell types develop senescence following irradiation, including microglia, astrocytes, and cerebrovascular endothelial cells [63, 64]. Among these senescent endothelial cells and microglia are particularly important sources of SASP factors [92], which can amplify neuroinflammation and promote systemic dissemination of pro-geronic signals. SASP components, such as pro-inflammatory cytokines, can enter the systemic circulation and influence distant tissues. Chronic low-grade inflammation is a well-recognized driver of sarcopenia promoting anabolic resistance, mitochondrial dysfunction, and activation of catabolic signaling pathways in muscle [41], [93–97]. Consistent with this framework, our findings support a model in which brain-derived inflammatory mediators contribute to a systemic pro-geronic environment that accelerates skeletal muscle aging.

Previous studies using the same fractionated WBI paradigm have demonstrated that cellular senescence in the brain develops rapidly and persists long-term [63–65]. Increased expression of senescence markers such as p16^*Ink4a*^, accumulation of senescent endothelial cells and astrocytes, and sustained elevation of SASP factors have been observed within weeks after irradiation and remain elevated for months [63–65]. Importantly, pharmacological clearance of senescent cells attenuates irradiation-induced cerebrovascular dysfunction, supporting a causal role for senescence rather than transient injury responses [63–65]. These observations suggest that brain senescence precedes and likely contributes to the development of systemic aging phenotypes. Future longitudinal studies, particularly those incorporating senolytic interventions, will be necessary to further define the temporal cascade linking brain senescence to peripheral tissue aging.

In addition to inflammatory signaling, disruption of neuroendocrine homeostasis likely contributes to the observed muscle phenotype. Skeletal muscle mass and function are tightly regulated by anabolic hormones, particularly IGF-1, which supports protein synthesis, mitochondrial biogenesis, and muscle regeneration. Both aging [38, 98–101] and cranial irradiation [48, 49, 52, 54] are associated with suppression of hypothalamic-pituitary signaling and reduced circulating IGF-1 levels. Importantly, the impact of brain irradiation on neuroendocrine function may arise through both direct and indirect mechanisms. Direct effects may result from radiation-induced damage and senescence within hypothalamic and pituitary regulatory circuits that control growth hormone release, whereas indirect effects may be mediated by chronic neuroinflammation and SASP factors that disrupt neuroendocrine signaling in a sustained manner. Clinical studies of cancer survivors exposed to cranial irradiation document long-term endocrine dysfunction alongside increased risk of frailty, muscle weakness, and reduced physical performance. Although circulating hormones were not measured in the present study, the activation of catabolic transcriptional regulators and suppression of metabolic pathways in skeletal muscle are consistent with reduced anabolic signaling. Collectively, these observations support a model in which brain senescence promotes skeletal muscle aging through a dual mechanism involving inflammatory SASP signaling and impaired neuroendocrine support.

Although acute neuroendocrine stress responses following irradiation cannot be completely excluded, several lines of evidence argue against transient stress as the primary driver of the observed muscle phenotype. Irradiation was restricted to the brain using precise collimation, preventing direct exposure of peripheral tissues. Moreover, transcriptomic alterations were assessed 2 months after irradiation, well beyond the timeframe of acute stress responses, and exhibited strong concordance with physiological aging signatures. These findings support a model in which persistent senescence-associated systemic signaling, rather than transient stress, mediates the observed skeletal muscle aging phenotype.

In addition to inflammatory and neuroendocrine signaling, emerging evidence suggests that circulating metabolites, immune mediators, and extracellular vesicles contribute to the systemic propagation of aging phenotypes [7]. These circulating factors function as mediators of inter-organ communication and may represent important components of the brain–muscle aging axis. These systemic drivers of aging and their roles in coordinating organismal aging have been extensively reviewed recently, highlighting the importance of circulating pro-geronic and anti-geronic factors in mediating peripheral tissue aging  [7, 68, 102].

The clinical implications of these findings are significant. Sarcopenia is a major determinant of frailty, disability, and loss of independence in older adults [1]. Our results suggest that accelerated brain aging, whether driven by lifestyle factors, chronic stress, metabolic disease, or therapeutic interventions such as cranial irradiation, may amplify skeletal muscle decline via systemic mechanisms. This may be particularly relevant for cancer survivors, in whom brain-directed therapies induce long-lasting senescence and neuroinflammation. Recognizing brain senescence as an upstream driver of muscle aging highlights the need for long-term monitoring of muscle health and physical function in these populations and raises the possibility that targeting brain senescence could mitigate peripheral frailty.

Several limitations of this study should be acknowledged. First, our analysis was based on bulk transcriptomic profiling, which does not allow resolution of cell type–specific responses within skeletal muscle. The observed transcriptional changes likely reflect contributions from multiple cell populations within skeletal muscle, including myofibers, satellite cells, endothelial cells, fibro-adipogenic progenitors, and infiltrating immune cells. The prominent suppression of mitochondrial and metabolic pathways suggests that myofibers are major contributors; however, vascular and inflammatory cells may also play important roles. Future studies employing single-cell transcriptomics and spatial profiling approaches will be required to define cell type-specific responses. Second, we did not directly assess muscle mass, fiber composition, contractile function, or exercise performance. While the observed transcriptional changes are strongly suggestive of sarcopenia-like remodeling, future studies will directly evaluate muscle mass, strength, contractile properties, and exercise capacity to establish the functional consequences of brain-induced skeletal muscle aging. Third, circulating inflammatory and endocrine factors were not measured in this pilot study. Identifying specific mediators of the brain–muscle signaling axis will be an important goal of future work. Fourth, neuromuscular junction dysfunction may also contribute to skeletal muscle aging in this model. Although neuromuscular junction-specific pathways were not among the most strongly altered in our analysis, subtle changes in pathways related to neuronal signaling and muscle structural remodeling were observed. Future studies specifically evaluating neuromuscular junction integrity will be important to further elucidate brain-muscle communication mechanisms.

Despite these limitations, the present study establishes brain senescence as a potent driver of aging-like molecular remodeling in skeletal muscle. Together with our complementary findings in the heart obtained using the same experimental cohort and irradiation paradigm, which demonstrated accelerated cardiac aging driven by brain senescence [63], these data support a model in which the aging brain functions as a central orchestrator of systemic aging across multiple organs. Interventions that target senescent brain cells, modulate neuroinflammatory signaling, or restore neuroendocrine balance may therefore represent promising strategies to preserve skeletal muscle health, delay frailty, and extend healthspan.

## References

[1] Sayer AA, Cooper R, Arai H, Cawthon PM, Ntsama Essomba MJ, Fielding RA, et al. Sarcopenia. Nat Rev Dis Primers. 2024;10:68. 10.1038/s41572-024-00550-w. 39300120 10.1038/s41572-024-00550-w

[2] Domaniku A, Bilgic SN, Kir S. Muscle wasting: emerging pathways and potential drug targets. Trends Pharmacol Sci. 2023;44:705–18. 10.1016/j.tips.2023.07.006. 37596181 10.1016/j.tips.2023.07.006

[3] Nunes-Pinto M, de Banira Mello RG, Pinto MN, Moro C, Vellas B, Martinez LO, et al. Sarcopenia and the biological determinants of aging: a narrative review from a geroscience perspective. Ageing Res Rev. 2025;103:102587. 10.1016/j.arr.2024.102587. 39571617 10.1016/j.arr.2024.102587

[4] Marzetti E, Calvani R, Coelho-Junior HJ, Landi F, Picca A. Mitochondrial quantity and quality in age-related sarcopenia. Int J Mol Sci. 2024. 10.3390/ijms25042052. 38396729 10.3390/ijms25042052 PMC10889427

[5] Can B, Kara O, Kizilarslanoglu MC, Arik G, Aycicek GS, Sumer F, et al. Serum markers of inflammation and oxidative stress in sarcopenia. Aging Clin Exp Res. 2017;29:745–52. 10.1007/s40520-016-0626-2. 27571781 10.1007/s40520-016-0626-2

[6] Zou Y, Ye H, Xu Z, Yang Q, Zhu J, Li T, et al. Obesity, sarcopenia, sarcopenic obesity, and hypertension: mediating role of inflammation and insulin resistance. J Gerontol A Biol Sci Med Sci. 2025. 10.1093/gerona/glae284. 39918389 10.1093/gerona/glae284

[7] Gulej R, Patai R, Ungvari A, Kallai A, Tarantini S, Yabluchanskiy A, et al. Impacts of systemic milieu on cerebrovascular and brain aging: insights from heterochronic parabiosis, blood exchange, and plasma transfer experiments. Geroscience. 2025. 10.1007/s11357-025-01657-y. 40407975 10.1007/s11357-025-01657-y PMC12635022

[8] Gonzalez-Armenta JL, Li N, Lee RL, Lu B, Molina AJA. Heterochronic parabiosis: old blood induces changes in mitochondrial structure and function of young mice. J Gerontol A Biol Sci Med Sci. 2021;76:434–9. 10.1093/gerona/glaa299. 33377482 10.1093/gerona/glaa299 PMC8177798

[9] Josephson AM, Leclerc K, Remark LH, Lopez EM, Leucht P. Systemic NF-κB-mediated inflammation promotes an aging phenotype in skeletal stem/progenitor cells. Aging Albany NY. 2021;13:13421–9. 10.18632/aging.203083. 34035186 10.18632/aging.203083 PMC8202837

[10] Sinha M, Jang YC, Oh J, Khong D, Wu EY, Manohar R, et al. Restoring systemic GDF11 levels reverses age-related dysfunction in mouse skeletal muscle. Science. 2014;344:649–52. 10.1126/science.1251152. 24797481 10.1126/science.1251152 PMC4104429

[11] Baht GS, Bareja A, Lee DE, Rao RR, Huang R, Huebner JL, et al. Meteorin-like facilitates skeletal muscle repair through a Stat3/IGF-1 mechanism. Nat Metab. 2020;2:278–89. 10.1038/s42255-020-0184-y. 32694780 10.1038/s42255-020-0184-y PMC7504545

[12] Gulej R, Nagy D, Kristof R, Csiszar A, Patai R. Circulating factors as modifiable therapeutic targets in brain and cerebrovascular aging: insights from heterochronic parabiosis. Adv Transl Res. 2026. 10.1556/1661.2025.00107.

[13] Xiao YZ, Yang M, Xiao Y, Guo Q, Huang Y, Li CJ, et al. Reducing hypothalamic stem cell senescence protects against aging-associated physiological decline. Cell Metab. 2020;31:534-548 e535. 10.1016/j.cmet.2020.01.002. 32004475 10.1016/j.cmet.2020.01.002

[14] Imai SI. The NAD world 2.0: the importance of the inter-tissue communication mediated by NAMPT/NAD(+)/SIRT1 in mammalian aging and longevity control. NPJ Syst Biol Appl. 2016;2:16018. 10.1038/npjsba.2016.18. 28725474 10.1038/npjsba.2016.18 PMC5516857

[15] Satoh A, Imai S. Systemic regulation of mammalian ageing and longevity by brain sirtuins. Nat Commun. 2014;5:4211. 10.1038/ncomms5211. 24967620 10.1038/ncomms5211 PMC4521907

[16] Zhang G, Li J, Purkayastha S, Tang Y, Zhang H, Yin Y, et al. Hypothalamic programming of systemic ageing involving IKK-beta, NF-kappaB and GnRH. Nature. 2013;497:211–6. 10.1038/nature12143. 23636330 10.1038/nature12143 PMC3756938

[17] Ungvari Z, Csiszar A. The emerging role of IGF-1 deficiency in cardiovascular aging: recent advances. J Gerontol A Biol Sci Med Sci. 2012;67:599–610. 10.1093/gerona/gls072. 22451468 10.1093/gerona/gls072 PMC3348495

[18] Jin K, Yao Z, van Velthoven CTJ, Kaplan ES, Glattfelder K, Barlow ST, et al. Brain-wide cell-type-specific transcriptomic signatures of healthy ageing in mice. Nature. 2025;638:182–96. 10.1038/s41586-024-08350-8. 39743592 10.1038/s41586-024-08350-8 PMC11798837

[19] Cai D, Khor S. Hypothalamic microinflammation. Handb Clin Neurol. 2021;181:311–22. 10.1016/B978-0-12-820683-6.00023-3. 34238467 10.1016/B978-0-12-820683-6.00023-3

[20] Bhusal A, Rahman MH, Suk K. Hypothalamic inflammation in metabolic disorders and aging. Cell Mol Life Sci. 2021;79:32. 10.1007/s00018-021-04019-x. 34910246 10.1007/s00018-021-04019-x PMC11071926

[21] Cai D, Khor S. “Hypothalamic microinflammation” paradigm in aging and metabolic diseases. Cell Metab. 2019;30:19–35. 10.1016/j.cmet.2019.05.021. 31269425 10.1016/j.cmet.2019.05.021

[22] Cai D, Liu T. Inflammatory cause of metabolic syndrome via brain stress and NF-kappaB. Aging Albany NY. 2012;4:98–115. 10.18632/aging.100431. 22328600 10.18632/aging.100431 PMC3314172

[23] Tang Y, Cai D. Hypothalamic inflammation and GnRH in aging development. Cell Cycle. 2013;12:2711–2. 10.4161/cc.26054. 23966154 10.4161/cc.26054 PMC3899179

[24] Zhang Y, Kim MS, Jia B, Yan J, Zuniga-Hertz JP, Han C, et al. Hypothalamic stem cells control ageing speed partly through exosomal miRNAs. Nature. 2017;548:52–7. 10.1038/nature23282. 28746310 10.1038/nature23282 PMC5999038

[25] Yang S, Tian M, Dai Y, Wang R, Yamada S, Feng S, et al. Infection and chronic disease activate a systemic brain-muscle signaling axis. Sci Immunol. 2024;9:eadm7908. 10.1126/sciimmunol.adm7908. 38996009 10.1126/sciimmunol.adm7908

[26] Bowman GL, Dayon L, Kirkland R, Wojcik J, Peyratout G, Severin IC, et al. Blood-brain barrier breakdown, neuroinflammation, and cognitive decline in older adults. Alzheimers Dement. 2018;14:1640–50. 10.1016/j.jalz.2018.06.2857. 30120040 10.1016/j.jalz.2018.06.2857

[27] Sweeney MD, Sagare AP, Zlokovic BV. Blood-brain barrier breakdown in Alzheimer disease and other neurodegenerative disorders. Nat Rev Neurol. 2018;14:133–50. 10.1038/nrneurol.2017.188. 29377008 10.1038/nrneurol.2017.188 PMC5829048

[28] Giovannini S, Marzetti E, Borst SE, Leeuwenburgh C. Modulation of GH/IGF-1 axis: potential strategies to counteract sarcopenia in older adults. Mech Ageing Dev. 2008;129:593–601. 10.1016/j.mad.2008.08.001. 18762207 10.1016/j.mad.2008.08.001 PMC5992490

[29] Everitt AV. The neuroendocrine system and aging. Gerontology. 1980;26:108–19. 10.1159/000212403. 6101319 10.1159/000212403

[30] Csik B, Nyul-Toth A, Gulej R, Patai R, Kiss T, Delfavero J, et al. Senescent endothelial cells in cerebral microcirculation are key drivers of age-related blood-brain barrier disruption, microvascular rarefaction, and neurovascular coupling impairment in mice. Aging Cell. 2025;e70048. 10.1111/acel.70048. 40167015 10.1111/acel.70048 PMC12266767

[31] Baker DJ, Petersen RC. Cellular senescence in brain aging and neurodegenerative diseases: evidence and perspectives. J Clin Invest. 2018;128:1208–16. 10.1172/JCI95145. 29457783 10.1172/JCI95145 PMC5873891

[32] Chinta SJ, Woods G, Rane A, Demaria M, Campisi J, Andersen JK. Cellular senescence and the aging brain. Exp Gerontol. 2014. S0531-5565(14)00275-7[pii]10.1016/j.exger.2014.09.018. 10.1016/j.exger.2014.09.018 PMC4382436 25281806

[33] Kiss T, Nyul-Toth A, Balasubramanian P, Tarantini S, Ahire C, DelFavero J, et al. Single-cell RNA sequencing identifies senescent cerebromicrovascular endothelial cells in the aged mouse brain. Geroscience. 2020. 10.1007/s11357-020-00177-1. 32236824 10.1007/s11357-020-00177-1 PMC7205992

[34] Kiss T, Nyul-Toth A, DelFavero J, Balasubramanian P, Tarantini S, Faakye J, et al. Spatial transcriptomic analysis reveals inflammatory foci defined by senescent cells in the white matter, hippocampi and cortical grey matter in the aged mouse brain. Geroscience. 2022;44:661–81. 10.1007/s11357-022-00521-7. 35098444 10.1007/s11357-022-00521-7 PMC9135953

[35] Tarantini S, Balasubramanian P, Delfavero J, Csipo T, Yabluchanskiy A, Kiss T, et al. Treatment with the BCL-2/BCL-xL inhibitor senolytic drug ABT263/Navitoclax improves functional hyperemia in aged mice. Geroscience. 2021;43:2427–40. 10.1007/s11357-021-00440-z. 34427858 10.1007/s11357-021-00440-z PMC8599595

[36] Bhardwaj G, Penniman CM, Klaus K, Weatherford ET, Pan H, Dreyfuss JM, et al. Transcriptomic regulation of muscle mitochondria and calcium signaling by insulin/IGF-1 receptors depends on FoxO transcription factors. Front Physiol. 2021;12:779121. 10.3389/fphys.2021.779121. 35185597 10.3389/fphys.2021.779121 PMC8855073

[37] Bhardwaj G, Penniman CM, Jena J, Suarez Beltran PA, Foster C, Poro K, et al. Insulin and IGF-1 receptors regulate complex I-dependent mitochondrial bioenergetics and supercomplexes via FoxOs in muscle. J Clin Invest. 2021. 10.1172/JCI146415. 10.1172/JCI146415 PMC8439595 34343133

[38] Ascenzi F, Barberi L, Dobrowolny G, Villa Nova Bacurau A, Nicoletti C, Rizzuto E, et al. Effects of IGF-1 isoforms on muscle growth and sarcopenia. Aging Cell. 2019;18:e12954. 10.1111/acel.12954. 30953403 10.1111/acel.12954 PMC6516183

[39] Bucci L, Yani SL, Fabbri C, Bijlsma AY, Maier AB, Meskers CG, et al. Circulating levels of adipokines and IGF-1 are associated with skeletal muscle strength of young and old healthy subjects. Biogerontology. 2013;14:261–72. 10.1007/s10522-013-9428-5. 23666343 10.1007/s10522-013-9428-5

[40] Scicchitano BM, Rizzuto E, Musaro A. Counteracting muscle wasting in aging and neuromuscular diseases: the critical role of IGF-1. Aging Albany NY. 2009;1:451–7. 10.18632/aging.100050. 20157530 10.18632/aging.100050 PMC2806025

[41] Pan L, Xie W, Fu X, Lu W, Jin H, Lai J, et al. Inflammation and sarcopenia: a focus on circulating inflammatory cytokines. Exp Gerontol. 2021;154:111544. 10.1016/j.exger.2021.111544. 34478826 10.1016/j.exger.2021.111544

[42] Roubenoff R, Parise H, Payette HA, Abad LW, D’Agostino R, Jacques PF, et al. Cytokines, insulin-like growth factor 1, sarcopenia, and mortality in very old community-dwelling men and women: the Framingham Heart Study. Am J Med. 2003;115:429–35. 14563498 10.1016/j.amjmed.2003.05.001

[43] Zhang H, Cherian R, Jin K. Systemic milieu and age-related deterioration. Geroscience. 2019;41:275–84. 10.1007/s11357-019-00075-1. 31152364 10.1007/s11357-019-00075-1 PMC6702503

[44] Rebo J, Mehdipour M, Gathwala R, Causey K, Liu Y, Conboy MJ, et al. A single heterochronic blood exchange reveals rapid inhibition of multiple tissues by old blood. Nat Commun. 2016;7:13363. 10.1038/ncomms13363. 27874859 10.1038/ncomms13363 PMC5121415

[45] Conboy IM, Conboy MJ, Wagers AJ, Girma ER, Weissman IL, Rando TA. Rejuvenation of aged progenitor cells by exposure to a young systemic environment. Nature. 2005;433:760–4. 10.1038/nature03260. 15716955 10.1038/nature03260

[46] Kiss T, Nyul-Toth A, Gulej R, Tarantini S, Csipo T, Mukli P, et al. Old blood from heterochronic parabionts accelerates vascular aging in young mice: transcriptomic signature of pathologic smooth muscle remodeling. Geroscience. 2022;44:953–81. 10.1007/s11357-022-00519-1. 35124764 10.1007/s11357-022-00519-1 PMC9135944

[47] Gulej R, Nyul-Toth A, Csik B, Petersen B, Faakye J, Negri S, et al. Rejuvenation of cerebromicrovascular function in aged mice through heterochronic parabiosis: insights into neurovascular coupling and the impact of young blood factors. Geroscience. 2024;46:327–47. 10.1007/s11357-023-01039-2. 38123890 10.1007/s11357-023-01039-2 PMC10828280

[48] Achermann JC, Hindmarsh PC, Brook CG. The relationship between the growth hormone and insulin-like growth factor axis in long-term survivors of childhood brain tumours. Clin Endocrinol (Oxf). 1998;49:639–45. 10.1046/j.1365-2265.1998.00585.x. 10197080 10.1046/j.1365-2265.1998.00585.x

[49] Vilela MI, Serravite MO, Oliveira NB, de Brito PC, Ribeiro-Oliveira A Jr., Viana MB. Height deficit and impairment of the GH/IGF-1 axis in patients treated for acute lymphoblastic leukemia during childhood. Horm Res Paediatr. 2013;79:9–16. 10.1159/000343936. 10.1159/000343936 23306635

[50] Sfeir JG, Kittah NEN, Tamhane SU, Jasim S, Chemaitilly W, Cohen LE, et al. Diagnosis of GH deficiency as a late effect of radiotherapy in survivors of childhood cancers. J Clin Endocrinol Metab. 2018;103:2785–93. 10.1210/jc.2018-01204. 29982753 10.1210/jc.2018-01204

[51] Pollock NI, Cohen LE. Growth hormone deficiency and treatment in childhood cancer survivors. Front Endocrinol (Lausanne). 2021;12:745932. 10.3389/fendo.2021.745932. 34745010 10.3389/fendo.2021.745932 PMC8569790

[52] Patterson BC, Meacham LR. Growth hormone deficiency and growth hormone replacement in childhood cancer survivors. Front Horm Res. 2021;54:25–35. 10.1159/000515111. 33934095 10.1159/000515111

[53] Follin C, Wiebe T, Moell C, Erfurth EM. Moderate dose cranial radiotherapy causes central adrenal insufficiency in long-term survivors of childhood leukaemia. Pituitary. 2014;17:7–12. 10.1007/s11102-012-0459-8. 23283630 10.1007/s11102-012-0459-8

[54] Anttonen J, Remes T, Arikoski P, Lahteenmaki P, Arola M, Harila-Saari A, et al. Pre- and postdiagnosis growth failure, adult short stature, and untreated growth hormone deficiency in radiotherapy-treated long-term survivors of childhood brain tumor. PLoS One. 2022;17:e0274274. 10.1371/journal.pone.0274274. 36067205 10.1371/journal.pone.0274274 PMC9447887

[55] Steinmann D, Vordermark D, Gerstenberg W, Aschoff R, Gharbi N, Muller A, et al. Quality of life in patients with limited (1-3) brain metastases undergoing stereotactic or whole brain radiotherapy : a prospective study of the DEGRO QoL working group. Strahlenther Onkol. 2020;196:48–57. 10.1007/s00066-019-01506-w. 31418046 10.1007/s00066-019-01506-w

[56] Goodenough CG, Partin RE, Ness KK. Skeletal muscle and childhood cancer: where are we now and where we go from here. Aging Cancer. 2021;2:13–35. 10.1002/aac2.12027. 34541550 10.1002/aac2.12027 PMC8445321

[57] Ness KK, DeLany JP, Kaste SC, Mulrooney DA, Pui CH, Chemaitilly W, et al. Energy balance and fitness in adult survivors of childhood acute lymphoblastic leukemia. Blood. 2015;125:3411–9. 10.1182/blood-2015-01-621680. 25814529 10.1182/blood-2015-01-621680 PMC4447859

[58] Ness KK, Hudson MM, Pui CH, Green DM, Krull KR, Huang TT, et al. Neuromuscular impairments in adult survivors of childhood acute lymphoblastic leukemia: associations with physical performance and chemotherapy doses. Cancer. 2012;118:828–38. 10.1002/cncr.26337. 21766297 10.1002/cncr.26337 PMC3197897

[59] Tonorezos ES, Robien K, Eshelman-Kent D, Moskowitz CS, Church TS, Ross R, et al. Contribution of diet and physical activity to metabolic parameters among survivors of childhood leukemia. Cancer Causes Control. 2013;24:313–21. 10.1007/s10552-012-0116-6. 23187859 10.1007/s10552-012-0116-6 PMC3557541

[60] Boland AM, Gibson TM, Lu L, Kaste SC, DeLany JP, Partin RE, et al. Dietary protein intake and lean muscle mass in survivors of childhood acute lymphoblastic leukemia: report from the St. Jude lifetime cohort study. Phys Ther. 2016;96:1029–38. 10.2522/ptj.20150507. 26893509 10.2522/ptj.20150507 PMC4935785

[61] Ness KK, Armstrong GT, Kundu M, Wilson CL, Tchkonia T, Kirkland JL. Frailty in childhood cancer survivors. Cancer. 2015;121:1540–7. 10.1002/cncr.29211. 25529481 10.1002/cncr.29211 PMC4424063

[62] Hayek S, Gibson TM, Leisenring WM, Guida JL, Gramatges MM, Lupo PJ, et al. Prevalence and predictors of frailty in childhood cancer survivors and siblings: a report from the childhood cancer survivor study. J Clin Oncol. 2020;38:232–47. 10.1200/JCO.19.01226. 31800343 10.1200/JCO.19.01226 PMC6968796

[63] Gulej R, Patai R, Kiss T, Chandragiri SS, Ekambaram S, Nagaraja RY, et al. Irradiation-induced brain senescence accelerates cardiac aging via systemic mechanisms: insights from transcriptomic profiling. Geroscience. 2025. 10.1007/s11357-025-01953-7. 41139380 10.1007/s11357-025-01953-7 PMC12972343

[64] Yabluchanskiy A, Tarantini S, Balasubramanian P, Kiss T, Csipo T, Fulop GA, et al. Pharmacological or genetic depletion of senescent astrocytes prevents whole brain irradiation-induced impairment of neurovascular coupling responses protecting cognitive function in mice. Geroscience. 2020;42:409–28. 10.1007/s11357-020-00154-8. 31960269 10.1007/s11357-020-00154-8 PMC7205933

[65] Gulej R, Nyul-Toth A, Ahire C, DelFavero J, Balasubramanian P, Kiss T, et al. Elimination of senescent cells by treatment with Navitoclax/ABT263 reverses whole brain irradiation-induced blood-brain barrier disruption in the mouse brain. Geroscience. 2023;45:2983–3002. 10.1007/s11357-023-00870-x. 37642933 10.1007/s11357-023-00870-x PMC10643778

[66] Conboy MJ, Conboy IM, Rando TA. Heterochronic parabiosis: historical perspective and methodological considerations for studies of aging and longevity. Aging Cell. 2013;12:525–30. 10.1111/acel.12065. 23489470 10.1111/acel.12065 PMC4072458

[67] Mehdipour M, Amiri P, Liu C, DeCastro J, Kato C, Skinner CM, et al. Small-animal blood exchange is an emerging approach for systemic aging research. Nat Protoc. 2022;17:2469–93. 10.1038/s41596-022-00731-5. 35986217 10.1038/s41596-022-00731-5 PMC10035053

[68] Pamplona R, Jove M, Gomez J, Barja G. Whole organism aging: parabiosis, inflammaging, epigenetics, and peripheral and central aging clocks. The ARS of aging. Exp Gerontol. 2023;174:112137. 10.1016/j.exger.2023.112137. 36871903 10.1016/j.exger.2023.112137

[69] Yu G, Li F, Qin Y, Bo X, Wu Y, Wang S. GosemSim: an R package for measuring semantic similarity among GO terms and gene products. Bioinformatics. 2010;26:976–8. 10.1093/bioinformatics/btq064. 20179076 10.1093/bioinformatics/btq064

[70] Muller-Dott S, Tsirvouli E, Vazquez M, Ramirez Flores RO, Badia IMP, Fallegger R, et al. Expanding the coverage of regulons from high-confidence prior knowledge for accurate estimation of transcription factor activities. Nucleic Acids Res. 2023;51:10934–49. 10.1093/nar/gkad841. 37843125 10.1093/nar/gkad841 PMC10639077

[71] Murphy T, Thuret S. The systemic milieu as a mediator of dietary influence on stem cell function during ageing. Ageing Res Rev. 2015;19:53–64. 10.1016/j.arr.2014.11.004. 25481406 10.1016/j.arr.2014.11.004

[72] Coen PM, Musci RV, Hinkley JM, Miller BF. Mitochondria as a target for mitigating sarcopenia. Front Physiol. 2018;9:1883. 10.3389/fphys.2018.01883. 30687111 10.3389/fphys.2018.01883 PMC6335344

[73] Daussin FN, Boulanger E, Lancel S. From mitochondria to sarcopenia: role of inflammaging and RAGE-ligand axis implication. Exp Gerontol. 2021;146:111247. 10.1016/j.exger.2021.111247. 33484891 10.1016/j.exger.2021.111247

[74] Del Campo A, Contreras-Hernandez I, Castro-Sepulveda M, Campos CA, Figueroa R, Tevy MF, et al. Muscle function decline and mitochondria changes in middle age precede sarcopenia in mice. Aging (Albany NY). 2018;10:34–55. 10.18632/aging.101358. 29302020 10.18632/aging.101358 PMC5811241

[75] Drew B, Phaneuf S, Dirks A, Selman C, Gredilla R, Lezza A, et al. Effects of aging and caloric restriction on mitochondrial energy production in gastrocnemius muscle and heart. Am J Physiol Regul Integr Comp Physiol. 2003;284:R474-480. 12388443 10.1152/ajpregu.00455.2002

[76] Wiedmer P, Jung T, Castro JP, Pomatto LCD, Sun PY, Davies KJA, et al. Sarcopenia - molecular mechanisms and open questions. Ageing Res Rev. 2021;65:101200. 10.1016/j.arr.2020.101200. 33130247 10.1016/j.arr.2020.101200

[77] McFarlane C, Plummer E, Thomas M, Hennebry A, Ashby M, Ling N, et al. Myostatin induces cachexia by activating the ubiquitin proteolytic system through an NF-kappaB-independent, FoxO1-dependent mechanism. J Cell Physiol. 2006;209:501–14. 10.1002/jcp.20757. 16883577 10.1002/jcp.20757

[78] Yu X, Chen X, Wu W, Tang H, Su Y, Lian G, et al. Zinc alleviates diabetic muscle atrophy via modulation of the SIRT1/FoxO1 autophagy pathway through GPR39. J Cachexia Sarcopenia Muscle. 2025;16:e13771. 10.1002/jcsm.13771. 40026072 10.1002/jcsm.13771 PMC11873538

[79] Xu J, Li R, Workeneh B, Dong Y, Wang X, Hu Z. Transcription factor FoxO1, the dominant mediator of muscle wasting in chronic kidney disease, is inhibited by microRNA-486. Kidney Int. 2012;82:401–11. 10.1038/ki.2012.84. 22475820 10.1038/ki.2012.84 PMC3393843

[80] Kamei Y, Miura S, Suzuki M, Kai Y, Mizukami J, Taniguchi T, et al. Skeletal muscle FOXO1 (FKHR) transgenic mice have less skeletal muscle mass, down-regulated Type I (slow twitch/red muscle) fiber genes, and impaired glycemic control. J Biol Chem. 2004;279:41114–23. 10.1074/jbc.M400674200. 15272020 10.1074/jbc.M400674200

[81] Zhao J, Brault JJ, Schild A, Cao P, Sandri M, Schiaffino S, et al. FoxO3 coordinately activates protein degradation by the autophagic/lysosomal and proteasomal pathways in atrophying muscle cells. Cell Metab. 2007;6:472–83. 10.1016/j.cmet.2007.11.004. 18054316 10.1016/j.cmet.2007.11.004

[82] Wang J, Gao X, Ren D, Zhang M, Zhang P, Lu S, et al. Triptolide induces atrophy of myotubes by triggering IRS-1 degradation and activating the FoxO3 pathway. Toxicol In Vitro. 2020;65:104793. 10.1016/j.tiv.2020.104793. 32061799 10.1016/j.tiv.2020.104793

[83] Sandri M, Lin J, Handschin C, Yang W, Arany ZP, Lecker SH, et al. PGC-1α protects skeletal muscle from atrophy by suppressing FoxO3 action and atrophy-specific gene transcription. Proc Natl Acad Sci USA. 2006;103:16260–5. 10.1073/pnas.0607795103. 17053067 10.1073/pnas.0607795103 PMC1637570

[84] Ninfali C, Siles L, Darling DS, Postigo A. Regulation of muscle atrophy-related genes by the opposing transcriptional activities of ZEB1/CtBP and FOXO3. Nucleic Acids Res. 2018;46:10697–708. 10.1093/nar/gky835. 30304480 10.1093/nar/gky835 PMC6237734

[85] Mammucari C, Milan G, Romanello V, Masiero E, Rudolf R, Del Piccolo P, et al. FoxO3 controls autophagy in skeletal muscle in vivo. Cell Metab. 2007;6:458–71. 10.1016/j.cmet.2007.11.001. 18054315 10.1016/j.cmet.2007.11.001

[86] Hirata Y, Nomura K, Senga Y, Okada Y, Kobayashi K, Okamoto S, et al. Hyperglycemia induces skeletal muscle atrophy via a WWP1/KLF15 axis. JCI Insight. 2019. 10.1172/jci.insight.124952. 30830866 10.1172/jci.insight.124952 PMC6478420

[87] Hirata Y, Nomura K, Kato D, Tachibana Y, Niikura T, Uchiyama K, et al. A Piezo1/KLF15/IL-6 axis mediates immobilization-induced muscle atrophy. J Clin Invest. 2022;132:1–13. 10.1172/JCI154611. 35290243 10.1172/JCI154611 PMC9159676

[88] Fan L, Sweet DR, Prosdocimo DA, Vinayachandran V, Chan ER, Zhang R, et al. Muscle Krüppel-like factor 15 regulates lipid flux and systemic metabolic homeostasis. J Clin Invest. 2021. 10.1172/JCI139496. 33586679 10.1172/JCI139496 PMC7880311

[89] Ono Y, Saito M, Yoshihara I, Kondo Y, Sakamoto K, Sugiyama J, et al. Sepsis-associated skeletal muscle wasting is ameliorated by pharmacological inhibition of the STAT3 signaling pathway in mice. Sci Rep. 2026. 10.1038/s41598-026-35815-9. 41521250 10.1038/s41598-026-35815-9 PMC12876058

[90] Li C, Gu X, Zhu Z, Pan X, Fan M, Liu X, et al. Ruxolitinib alleviated muscle atrophy in cancer cachexia by inhibiting IL-6/JAK/STAT3 signaling pathway in mice. J Pharm Pharmacol. 2025;77:1715–25. 10.1093/jpp/rgaf073. 40834115 10.1093/jpp/rgaf073

[91] Kumar G, Khandibharad S, Singh S. Targeting IL-6/STAT3 signaling to mitigate sarcopenia: insights from immuno-metabolic crosstalk in NSCLC. Biochem Biophys Res Commun. 2025;786:152727. 10.1016/j.bbrc.2025.152727. 41038071 10.1016/j.bbrc.2025.152727

[92] Ungvari Z, Podlutsky A, Sosnowska D, Tucsek Z, Toth P, Deak F, et al. Ionizing radiation promotes the acquisition of a senescence-associated secretory phenotype and impairs angiogenic capacity in cerebromicrovascular endothelial cells: role of increased DNA damage and decreased DNA repair capacity in microvascular radiosensitivity. J Gerontol A Biol Sci Med Sci. 2013;68:1443–57. 10.1093/gerona/glt057. 23689827 10.1093/gerona/glt057 PMC3814240

[93] Cataltepe E, Ceker E, Fadiloglu A, Gungor F, Karakurt N, Ulger Z, et al. Association between the systemic immune-inflammation index and sarcopenia in older adults: a cross-sectional study. BMC Geriatr. 2025;25:28. 10.1186/s12877-025-05686-2. 39806294 10.1186/s12877-025-05686-2 PMC11727228

[94] Livshits G, Kalinkovich A. A cross-talk between sestrins, chronic inflammation and cellular senescence governs the development of age-associated sarcopenia and obesity. Ageing Res Rev. 2023;86:101852. 10.1016/j.arr.2023.101852. 36642190 10.1016/j.arr.2023.101852

[95] Lopes KG, Farinatti P, Bottino DA, de Souza M, Maranhao PA, Bouskela E, et al. Sarcopenia in the elderly versus microcirculation, inflammation status, and oxidative stress: a cross-sectional study. Clin Hemorheol Microcirc. 2022;80:185–95. 10.3233/CH-211202. 34511490 10.3233/CH-211202

[96] Kalinkovich A, Livshits G. Sarcopenic obesity or obese sarcopenia: a cross talk between age-associated adipose tissue and skeletal muscle inflammation as a main mechanism of the pathogenesis. Ageing Res Rev. 2017;35:200–21. 10.1016/j.arr.2016.09.008. 27702700 10.1016/j.arr.2016.09.008

[97] Bano G, Trevisan C, Carraro S, Solmi M, Luchini C, Stubbs B, et al. Inflammation and sarcopenia: a systematic review and meta-analysis. Maturitas. 2017;96:10–5. 10.1016/j.maturitas.2016.11.006. 28041587 10.1016/j.maturitas.2016.11.006

[98] Jiang JJ, Chen SM, Chen J, Wu L, Ye JT, Zhang Q. Serum IGF-1 levels are associated with sarcopenia in elderly men but not in elderly women. Aging Clin Exp Res. 2022;34:2465–71. 10.1007/s40520-022-02180-2. 35962897 10.1007/s40520-022-02180-2

[99] Wennberg AMV, Hagen CE, Petersen RC, Mielke MM. Trajectories of plasma IGF-1, IGFBP-3, and their ratio in the Mayo Clinic Study of Aging. Exp Gerontol. 2018;106:67–73. 10.1016/j.exger.2018.02.015. 29474865 10.1016/j.exger.2018.02.015 PMC5911407

[100] Bodart G, Goffinet L, Morrhaye G, Farhat K, de Saint-Hubert M, Debacq-Chainiaux F, et al. Somatotrope GHRH/GH/IGF-1 axis at the crossroads between immunosenescence and frailty. Ann N Y Acad Sci. 2015;1351:61–7. 10.1111/nyas.12857. 26284958 10.1111/nyas.12857

[101] Gong Z, Kennedy O, Sun H, Wu Y, Williams GA, Klein L, et al. Reductions in serum IGF-1 during aging impair health span. Aging Cell. 2014;13:408–18. 10.1111/acel.12188. 10.1111/acel.12188 PMC4326899 24341939

[102] Villeda SA, Wyss-Coray T. The circulatory systemic environment as a modulator of neurogenesis and brain aging. Autoimmun Rev. 2013;12:674–7. 10.1016/j.autrev.2012.10.014. 23201925 10.1016/j.autrev.2012.10.014

